# Cortical thickness abnormalities in long-term remitted cushing’s disease

**DOI:** 10.1192/j.eurpsy.2021.363

**Published:** 2021-08-13

**Authors:** S. Bauduin, Z. Van Der Pal, A. Pereira, O. Meijer, E. Giltay, N. Van Der Wee, S. Van Der Werff

**Affiliations:** 1 Department Of Psychiatry, Leiden University Medical Center, Leiden, Netherlands; 2 Department Of Endocrinology, Leiden University Medical Center, Leiden, Netherlands

**Keywords:** Neuroimaging, Cushing’s Disease, endocrinology, Psychiatric symptomatology

## Abstract

**Introduction:**

Remitted Cushing’s disease (RCD)-patients commonly continue to present persistent psychological and cognitive deficits, and alterations in brain function and structure. Assessing cortical thickness and surface area of RCD-patients may offer further insight into the neuroanatomical substrates of Cushing’s disease.

**Objectives:**

To assess cortical thickness and surface area in RCD-patients in comparison to healthy controls (HCs).

**Methods:**

Structural 3T MRI’s were obtained from 25 long-term RCD-patients, and 25 age-, gender-, and education-matched HCs. T1-weighted images were segmented to extract mean cortical thickness and surface area values of 68 cortical gray matter regions. Paired sample t-tests explored differences between the anterior cingulate cortex (ACC; region of interest), and the whole brain. Validated scales assessed psychiatric symptomatology, self-reported cognitive functioning, and disease severity.

**Results:**

After correction for multiple comparisons, ROI analyses indicated that RCD-patients showed reduced cortical thickness of the left caudal ACC and the right rostral ACC compared to HCs. Whole-brain analyses indicated thinner cortices of the left caudal ACC, left cuneus, left posterior cingulate cortex, right rostral ACC, and bilateral precuneus compared to HCs. No cortical surface area differences were identified. Cortical thickness of the left caudal ACC was inversely associated with anxiety symptoms and disease duration.

**Conclusions:**

In six of 68 regions examined, RCD patients had reduced cortical thickness in comparison to HCs. Cortical thickness of the left caudal ACC was inversely associated with disease duration, suggesting that prolonged and excessive exposure to glucocorticoids may be related to cortical thinning of brain structures involved in emotional and cognitive processing.

**Disclosure:**

No significant relationships.

